# Amalgam Fillings

**Published:** 1883-06

**Authors:** 


ARTICLE VI

### AMALGAM FILLINGS.

Much has been said and written, pro and con, relating to amalgam as a filling material. We do not propose to further discuss the question with regard to its usefulness in saving teeth, or the advisability of employing it in many cases which the dentist is called upon to treat.

It is conceded by a vast majority of the very best men in the profession that there are cases in which its skillful and intelligent use gives more satisfaction than can be obtained with any other material yet known to dentisty. We simply wish to enter our protest against the careless and slovenly manner in which it is frequently used, even by some of our best manipulators of gold.

We have seen mouths containing gold fillings whose artistic contours, and beautiful finish indicated a high degree of manipulative skill in that direction ; and in the same mouth, amalgam fillings with rough surface, imperfect and often over-hanging edges (and the whole appearance of which was a disgrace to any dentist,) all put in by the same operator, of whose ability as a *dentist* we have no better opinion than we have of the man who can make a *good* amalgam filling, but whose operations in gold are not of the best.

It has been often said by those gentlemen who contend for the exclusive use of gold, that the failure of that material to produce invariably good results is due to defective manipulation. If this is true in regard to gold, it is no less true with regard to the material under consideration, and we believe that as large a percentage of its failures is due to the careless manner in which it is introduced and finished as is the case with any other material.

No one would expect satisfactory results from an imperfectly adapted, poorly finished gold filling, and we have not yet learned to expect more from amalgam when employed under like conditions.

That amalgam is most frequently indicated in teeth below the average in density, or in cavities of such extensive decay that the walls have become thin and friable, is no use for its hasty and unskilful employment. On the contrary, the poorer the tooth-structure, and the more delicate the walls of the cavity (or in the language of the New Departurists) "the more a tooth needs saving" the greater the care and skill required both in the preparation of the cavity and the introduction and finishing of the filling, in order to accomplish that end,—no matter of what material the filling may be composed.

Judging from the appearance of many fillings seen in the class of teeth just mentioned, it would seem that the operator has hardly deemed them worthy of any attention at all, and that he was evidently not a believer in the old adage, "what is worth doing at all is worth doing *well.*"

A dentist's business success should depend, primarily, upon his ability to *save teeth*, and his reputation in this direction may as frequently be enhanced by the good results produced with amalgam in some badly decayed, broken down molar, as by his skill in the manipulation of gold.

We are not a believer in the wholesale use of amalgam, but we do use it where, in our judgment, it is indicated and with the indulgence of our readers, will give the manner of using it which has given us the most satisfaction.

The cavity should be absolutely dry, carefully and thoroughly prepared,—in fact, when prepared it should be in as good condition as it would be if it were to receive a gold instead of an amalgam fillIng. The fillings should be well incorporated with just sufficient mercury to form a smooth, easy working mass; this is most quickly and thoroughly done in a small mortar. In introducing the amalgam into the cavity, small or moderately sized pieces should be used, and each piece carefully adapted to the walls of the cavity or incorporated with that which has preceded it, by tapping with properly shaped instruments as recommended by Prof. Flagg.

Should there be an excess of mercury, it must be removed from time to time during the progress of the filling until finally, when the cavity is full, all excess should be removed with pledgets of cotton, or, what we thnk better, by the use of wafers of amalgam from which all the mercury possible has been squeezed by the use of pliers and buckskin.

If the amalgam should be a quick setting variety, a fairly good finish may be obtained at a time of introduction, but we never feel satisfied until at a subsequent sitting, when the filling has become thoroughly hard, we have had an opportunity to give it a final polish. In doing this we never use a burnisher, for if there are delicate edges they would be liable to fracture.

With the lava strips, emery cloth, tape and fine pumice, or with some of the various polishing points for use with the dental engine, there is little difficulty in obtaining a beautiful surface, with the junction between the filling and cavity-edge absolutely flush and perfect.

We are satisfied that very many of the failures of amalgam fillings would be averted by more careful attention to this last step of the operation. If any of our readers have never tried this method of using amalgam, we advise them to do so. They will be astonished at the neater and cleaner appearance of the mouth, to say nothing of the increased durability of the fillings.—*Dental Practitioner.*

